# Risk factors for persistent abnormality on chest radiographs at 12-weeks post hospitalisation with PCR confirmed COVID-19

**DOI:** 10.1186/s12931-021-01750-8

**Published:** 2021-05-21

**Authors:** T. J. M. Wallis, E. Heiden, J. Horno, B. Welham, H. Burke, A. Freeman, L. Dexter, A. Fazleen, A. Kong, C. McQuitty, M. Watson, S. Poole, N. J. Brendish, T. W. Clark, T. M. A. Wilkinson, M. G. Jones, B. G. Marshall

**Affiliations:** 1 Department of Respiratory Medicine and Southampton NIHR Biomedical Research Centre, University Hospital Southampton and School of Clinical and Experimental Sciences, Faculty of Medicine, University of Southampton, Southampton, UK; 2 Department of Respiratory Medicine, University Hospital Southampton, Southampton, UK; 3 Department of Respiratory Medicine, University Hospital Southampton and School of Clinical and Experimental Sciences, Faculty of Medicine, University of Southampton, Southampton, UK; 4 Department of Infection and Southampton NIHR Biomedical Research Centre, University Hospital Southampton and School of Clinical and Experimental Sciences, Faculty of Medicine, University of Southampton, Southampton, UK; 5 Department of Infection, University Hospital Southampton and School of Clinical and Experimental Sciences, Faculty of Medicine, University of Southampton, Southampton, UK; 6 NIHR Southampton Biomedical Research Centre Research Fellow, University of Southampton, MP218 D-Level South Academic Block University Hospital Southampton, Southampton, SO16 6YD UK

**Keywords:** COVID-19, SARS-CoV-2, Follow-up study, Chest radiograph, LDH

## Abstract

**Background:**

The long-term consequences of COVID-19 remain unclear. There is concern a proportion of patients will progress to develop pulmonary fibrosis. We aimed to assess the temporal change in CXR infiltrates in a cohort of patients following hospitalisation for COVID-19.

**Methods:**

We conducted a single-centre prospective cohort study of patients admitted to University Hospital Southampton with confirmed SARS-CoV2 infection between 20th March and 3rd June 2020. Patients were approached for standard-of-care follow-up 12-weeks after hospitalisation. Inpatient and follow-up CXRs were scored by the assessing clinician for extent of pulmonary infiltrates; 0–4 per lung (Nil = 0, < 25% = 1, 25–50% = 2, 51–75% = 3, > 75% = 4).

**Results:**

101 patients with paired CXRs were included. Demographics: 53% male with a median (IQR) age 53.0 (45–63) years and length of stay 9 (5–17.5) days. The median CXR follow-up interval was 82 (77–86) days with median baseline and follow-up CXR scores of 4.0 (3–5) and 0.0 (0–1) respectively. 32% of patients had persistent CXR abnormality at 12-weeks. In multivariate analysis length of stay (LOS), smoking-status and obesity were identified as independent risk factors for persistent CXR abnormality. Serum LDH was significantly higher at baseline and at follow-up in patients with CXR abnormalities compared to those with resolution. A 5-point composite risk score (1-point each; LOS ≥ 15 days, Level 2/3 admission, LDH > 750 U/L, obesity and smoking-status) strongly predicted risk of persistent radiograph abnormality (0.81).

**Conclusion:**

Persistent CXR abnormality 12-weeks post COVID-19 was common in this cohort. LOS, obesity, increased serum LDH, and smoking-status were risk factors for radiograph abnormality. These findings require further prospective validation.

**Supplementary Information:**

The online version contains supplementary material available at 10.1186/s12931-021-01750-8.

## Introduction

The longer-term consequences of COVID-19, the disease caused by infection with severe acute respiratory syndrome coronavirus 2 (SARS-CoV-2), remain unclear. Long term progression to pulmonary fibrosis has previously been identified following infection with other species of the coronavirus family e.g., severe acute respiratory syndrome (SARS-CoV-1) and Middle East respiratory syndrome (MERS-CoV) [1]. Consequently, early in the COVID-19 pandemic, consensus guidelines based upon expert opinion were published proposing follow-up strategies for patients with COVID-19 to monitor patients for longer term complications [2, 3].

Short term follow-up studies of patients with COVID-19 have demonstrated that fibrotic changes in the lungs can be detected after as little as 3 weeks in patients across the whole spectrum of disease severity [4, 5]. Studies of medium-term follow-up are currently limited. In a real-world prospective follow-up study of COVID-19 survivors Mandal et al. identify persistent chest radiography abnormality in 38% of patients at a median follow-up interval of 54 days [6]. A prospective study of sixty COVID-19 survivors has identified ground-glass opacities and reticulation affecting greater than 10% lung volume on computed-tomography (CT) scanning in 55% of patients at 3-month follow-up [7]. Further in two retrospective studies, persistent CT abnormalities were identified in 42.3% [8] and 70% [9] of patients at 3-months. Zhao et al. [9] identify serum urea as a significant independent risk factor for persisting abnormal radiology at follow-up. Predicting patients who are at increased risk of long-term complications of COVID-19 is of clinical importance. It could help stratify patients at greater need of long-term follow-up and potentially assist in streamlining of follow-up services given the large number of patients who have been hospitalised due to COVID-19. Further identifying a phenotype of patients at high risk of long-term adverse outcome may facilitate targeted intervention with future disease directed therapies before the onset of complications.

We investigated temporal changes in chest radiograph appearances in a cohort of patients hospitalised with PCR confirmed COVID-19 undergoing standard of care follow up. Our aim was to investigate both the incidence of, and risk factors for, persistent COVID-19 related infiltrates at 12-week follow-up. A study exploring predictors of shorter-term clinical outcomes from a sub-group of patients in this cohort has been published previously [10].

## Methods

This was a prospective observational cohort study in a single academic medical centre University Hospital Southampton NHS Foundation Trust (UHSFT) of all patients undergoing 12-week virtual follow up clinic between 23rd June and 1st September 2020 following hospital admission with symptomatic SARS-CoV-2 infection. Ethical approval [REC reference (20/HRA/2986)] was obtained as part of the REACT observational study of COVID-19 (a longitudinal cohort study to facilitate better understanding and management of COVID-19). Informed consent was waived because of the study design. Data was collected through electronic case note review and using a real-time data analytics tool (REal-time Analytics for Clinical Trials [REACT]; digital Experimental Medicines Team, Manchester, UK).

### Patient selection

Consecutive patients were invited for clinic follow up by the clinical team and included all patients admitted to a Level 2 (High Dependency) or Intensive Care Facility (Level 3) at UHSFT between 20th March and 3rd June, all patients hospitalised between 1st May and 3^rd^ June 2020, and patients admitted between 20th March and 29th April 2020 who had consented to participate in the CoV-19POC research study [11], a trial assessing the clinical impact of molecular POCT in patients within 24 h of presentation to UHSFT and who had serum sampling performed.

All patients had PCR confirmed SARS-CoV-2 infection and were aged 18–90 years. PCR testing was performed by combined nose and throat swabs either using UHSFT standard of care laboratory SARS-CoV-2 testing or the CE marked QIAstat-Dx® Respiratory SARS-CoV Panel (Qiagen™, Manchester, UK) as part of the COV-19POC study as previously described [11].

Patients who were discharged to a nursing home, had severe dementia or who had metastatic malignancy with less than 1 year predicted survival as judged by the clinical team were excluded from follow-up.

### 12-week follow-up

All patients identified as appropriate for outpatient follow-up were invited to attend for a chest radiograph and blood tests as part of their standard clinical care at the 12-week timepoint following the date of their admission to hospital. Virtual follow-up appointments were then completed via telephone between 23rd June and 1st September 2020.

### Radiological severity scoring

Baseline and 12-week follow-up chest radiographs were scored for severity by the assessing clinician at the virtual follow-up appointment. A score of 0–4 was given for each lung dependent on the extent of pulmonary infiltrates (0 = No involvement, 1 ≤ 25%, 2 = 25–50%, 3 = 51–75%, 4 ≥ 75%) following the methodology of Wong et al. [12] giving a total score ranging from 0 to 8.

The baseline chest radiographs were defined as the last film prior to patient’s discharge (or the admission film if only one radiograph was performed). This was either a posteroanterior (PA) chest radiograph or anteroposterior (AP) film dependent on which film type had been performed as standard of care. Follow-up outpatient films were all departmental PA chest radiographs.

### Pathology

Baseline and 12-week bloods were taken as part of standard clinical care and analysed according to local validated clinical pathology standard operating procedures at UHSFT.

### Statistical methods

Initial statistics were descriptive in nature with baseline characteristics presented as frequency and percentages for categorical variables and median and inter-quartile range (IQR) for continuous variables. Between group comparisons for continuous variables were made using the Mann–Whitney U test. Spearman’s correlation coefficients were used to quantify correlations between continuous variables. Assessment of difference in distribution of categorical variables between independent groups was made using the Chi-squared test (χ^2^) or Fisher’s Exact test as appropriate. Time to event analysis was computed using Cox regression analysis. Variables identified with a p-value of < 0.2 in univariate analysis were selected for inclusion in a combined multivariate analysis. Model discrimination is presented using the area under the receiver operating characteristic curve (AUROC). p values of < 0.05 were deemed significant. Statistical analysis was conducted using IBM®-SPSS® (version 26) and *Graphpad* Prism (Version 8.4.3).

## Results

### Baseline demographics

A total of 134 patients were invited for follow-up (10 patients declined follow-up, 13 patients were lost to follow-up and 10 patients did not attend for chest radiographs). Therefore 101 patients who had complete data including paired baseline and follow-up chest radiographs were included in the final analysis (study CONSORT diagram see Additional File 1: Figure S1).

Baseline demographics for the cohort of 101 patients (Table 1) identified 53.5% were males with a median (IQR) age of 53.0 (45–63) years. The median baseline to follow-up chest radiograph interval, and admission to virtual follow-up appointment, was 82 (77–86) days and 96 (94–98) days respectively. The median length of stay was 9 (5–17.5) days and 48.5% (n = 49) of patients were admitted to a level 2 or 3 care facility. The majority of patients were white British ethnicity (65%) and never smokers (65%). Common comorbities included hypertension (36%), obesity [BMI > 30 kg/m^2^] (28%) and diabetes melitus [all types] (15%).

**Table 1:** Cohort baseline characteristics for the whole group (n = 101) and comparing those with complete Chest radiograph resolution (n = 69) vs. those with persistent abnormality (n = 32)

Variable	Whole Group (n = 101)	Complete Resolution (n = 69)	Persistent CXR Abnormality (n = 32)	p value
Sex (male)	53.5% (n = 54)	52.2% (n = 36)	56.3% (n = 18)	0.702
Age (years)	53.0 (45–63)	52.0 (42.5–62.5)	57.0 (50.3–63.8)	0.132
Length of Stay (days)	9 days (5–17.5)	8.0 (3–10.5)	20.5 (8.3–32.5)	0.001**
Follow-up CXR interval (days)	82 (77–86)	83.0 (76.0–90.0)	81.5 (65—87.5)	0.371
Admission to follow-up interval (days)	96 (94–98)	97.0 (94–112.5)	110.5 (95.2–126.0)	0.107
Level 2 or 3 Care	48.5% (n = 49)	39.1% (n = 27)	68.8% (n = 22)	0.006**
IMV	29.7% (n = 30)	21.7% (n = 15)	46.9% (n = 15)	0.010*
CPAP/NIV	18.8% (n = 19)	17.4% (n = 12)	21.9% (n = 7)	0.592
ECMO	3.0% (n = 3)	1.4% (n = 1)	6.3% (n = 2)	0.235
Oxygen or higher respiratory support	83% (n = 84)	81.2% (n = 56)	87.5% (n = 28)	0.428
BAME	35% (n = 35)	34.8% (n = 24)	34.4% (n = 11)	0.985
Obesity (BMI > 30 kg/m^2^)	28% (n = 28)	23.2% (n = 16)	34.4% (n = 11)	0.237
Hypertension	36% (n = 36)	31.9% (n = 22)	43.8% (n = 14)	0.247
Diabetes Melitus (all types)	18% (n = 19)	17.4% (n = 12)	21.9% (n = 7)	0.592
Asthma	15% (n = 15)	13% (n = 9)	18.8% (n = 6)	0.549
COPD	2% (n = 2)	0% (n = 0)	6% (n = 2)	0.098
Ischaemic Heart Disease	8% (n = 8)	5.8% (n = 4)	12.5% (n = 4)	0.259
Current or Ex-smoker	35% (n = 35)	24% (n = 16)	56% (n = 19)	0.002**

Values presented as Median (Interquartile range) for continuous variables and percentage (n) for categorical variables. CXR-chest radiograph, Level 2—High Dependancy Facility, Level 3-Intensive Care Facility, *IMV* invasive mechanical ventilation, *CPAP* continuous positive pressure ventilation, *NIV* non invasive ventilation, *ECMO* extracorporeal membrane oxygenation, *COPD* chronic obstructive pulmonary disease, *BAME* Black, Asian and Minority Ethnic, *BMI* body mass index (kg/m^2^)

*p < 0.05

**p < 0.01. Comparison of data for complete resolution vs. persistent CXR abnormality assessed using the Mann–Whitney U Test

### Clinical outcomes

At 12-week follow-up 65% (n = 65) of patients had one or more persisting symptom of which fatigue (41%) and breathlessness (38%) were the most common. 55% (n = 55) of patients were discharged following their 12-week virtual clinic. At the time of writing further investigations completed included; pulmonary function testing (in 36% of cohort) see Additional file 1: Table S1, transthoracic echocardiogram (43%), CT Chest (25%), repeat blood tests (8%), and repeat chest radiograph (3%).

### *Chest* radiograph scoring

The median (IQR) baseline chest radiograph score was 4.0 (3–5) with a maximum score of 8. At follow-up the median (IQR) chest radiograph severity score was 0 (0–1) with a maximum of 7. Overall 31.6% (n = 32) of patients had persistent chest radiograph changes at follow-up. Of those with persistent chest radiograph change, 55% (n = 17) had an infiltrate score of 1, 22% (n = 7) had a score of 2, and 23% (n = 8) had a score of 3 or greater. There was no significant difference in the median chest radiograph follow-up interval between those with resolution and persistent radiograph abnormality; 83.0 days (76.0–99.0) vs. 81.5 days (65.5–87.5) respectively, p = 0.37. At the time of writing, 14 patients with persistent chest radiograph abnormality have proceeded to have a chest CT scan, 86% (n = 12) of which have identified evidence of pulmonary fibrosis (for description see Additional file 1).

### Predicting persistent chest radiograph abnormality

Patients with abnormal chest radiographs at follow-up had a significantly longer median length of stay (20.5 days vs. 8.0 days p < 0.01) and were more likely to be current or previous smokers compared to never smokers (56% vs. 23% p = 0.02). Furthermore, patients with persistent radiograph abnormality were more likely to have been admitted to a level 2 or 3 care facility compared to those with complete radiograph resolution (45% vs. 19%) p < 0.01. Correlation was observed between total inpatient and follow-up chest radiograph scores r = 0.340 p = 0.001. There was no significant difference between groups in the distribution in the use of oxygen, by past medical history, ethnicity or in the follow-up interval between inpatient and follow-up chest radiographs (Table 1).

Univariate regression analysis identified length of stay, age, and current or ex-smoker status as significant risk factors for persistent chest radiograph change (Table 2). In multivariate analysis (covariates; length of stay, age at admission, level 2 or 3 admission, current or ex-smoking status and obesity) length of stay, current or ex-smoking status and obesity were identified as independent risk factors for persistent chest radiograph abnormality (Table 3).

**Table 2:** Cox-univariate analysis for risk of persistent chest radiograph (CXR) abnormality

Variable	Univariate analysis: Hazard Ratio (95% confidence interval)	p value
Age (years)	1.029 (1.001–1.057)	0.045*
Sex (male)	2.325 (0.658–2.702)	0.462
Length of Stay (days)	1.040 (1.022–1.059)	0.001**
Level 2 or 3 admission	1.847 (0.847–4.025)	0.123^+^
Oxygen or higher respiratory support	1.420 (0.495–4.077)	0.514
Current or ex-smoker	2.515 (1.225–5.165)	0.012*
BAME	0.675 (0.318–1.434)	0.307
Asthma	1.223 (0.503–2.977)	0.657
Hypertension	1.218 (0.603–2.460)	0.582
Obesity (BMI > 30 kg/m^2^)	1.762 (0.847–3.665)	0.129^+^
Diabetes Melitus	1.135 (0.488–2.639)	0.769

Variables with p < 0.2 in univariate analysis were included in the multivariate composite analysis. Level 2—High Dependancy Facility, Level 3-Intensive Care Facility, *BAME* Black, Asian and Minority Ethnic, *BMI* body mass index kg/m^2^

*p < 0.05

**p < 0.01

^+^p < 0.2

**Table 3:** Cox multivaraite analysis for risk of persistent CXR abnormality

Variable	Multivariate analysis Hazard Ratio (95% Confidence interval)	p value
Length of Stay (days)	1.060 (1.032–1.090)	< 0.001**
Age (years)	1.008 (0.970–1.047)	0.684
Current or ex-smoker	3.286 (1.352–7.982)	0.009**
Level 2 or 3 admission	0.825 (0.307–2.215)	0.702
Obesity (BMI > 30 kg/m^2^)	2.717 (1.144–6.454)	0.024*

Variables with p < 0.2 in univariate analysis were included in the multivariate composite analysis. Level 2—High Dependancy Facility, Level 3-Intensive Care Facility, *BAME* Black, Asian and Minority Ethnic, *BMI* body mass index kg/m^2^

*p < 0.05

**p < 0.01

^+^p < 0.2

### Analysis of laboratory indices

**Fig. 1:**
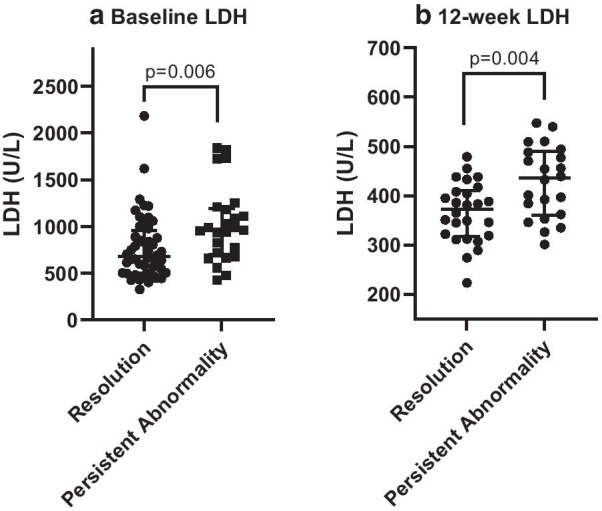
**a, b** Comparison of baseline and 12-week follow-up Serum Lactate Dehydrogenase (LDH) values (U/L) for patients with complete chest radiograph resolution (Resolution) vs. patients with persistent chest radiograph abnormality (Persistent Abnormality). Individual data points presented with error bars representing them median and upper and lower interquartile range. **a** illustrates baseline data and **b** illustrates 12-week follow-up data. p values assessed using Mann–Whitney U Test

Baseline and follow-up pathology data are summarised in Table 4. This analysis identified that serum lactate dehydrogenase (LDH) was the only blood marker significantly elevated in patients with persistent chest radiograph abnormality compared to those with complete resolution at both baseline and follow-up; median (IQR) 959 U/L (697–1193) vs. 679 U/L (502–955) p = 0.006 (n = 76) and 435 U/L (359–489) vs. 373 U/L (317–405) p = 0.004 (n = 46) respectively Fig. 1a, b. Further analysis of LDH results identified significant correlation between baseline serum LDH levels and both baseline and follow-up chest radiograph scores (r = 0.34, p = 0.003 and r = 0.34 p = 0.003 respectively). Follow-up serum LDH levels correlated significantly with follow-up chest radiograph scores (r = 0.39 p = 0.007). The baseline serum LDH was also significantly correlated with length of stay (r = 0.43 p < 0.001).

**Table 4:** Baseline and Follow-up Pathology Data for patients reviewed in the 12-week post hospitilisation COVID-19 virtual clinic

Variable	Baseline Pathology Results				12-week Pathology Results			
	n	Complete Resolution	Persistent Abnormality	p value	n	Complete Resolution	Persistent Abnormality	p value
Hb (g/L)	98	132 (118–143)	136 (126–146)	0.159	97	140 (131–147)	140 (124–147)	0.793
WBC (10 × 9/L)	98	6.7 (5.0–8.3)	6.8 (4.6–11.4)	0.616	97	7.0 (4.5–6.6)	7.3 (6.1–9.3)	0.190
Neutrophils (10 × 9/L)	98	4.8 (3.6–6.3)	5.7 (3.7–8.8)	0.296	97	3.7 (2.6–4.4)	4.0 (3.1–5.2)	0.164
Lymphocytes (10 × 9/L)	98	1.0 (0.8–1.2)	1.0 (0.7–1.5)	0.882	97	2.0 (1.6–2.7)	2.4 (1.8–3.0)	0.163
Sodium (mmol/L)	96	137 (135–140)	135 (133–138)	0.007**	97	140 (139–142)	141 (139–142)	0.752
Urea (mmol/L)	96	4.8 (3.6–6.2)	6.4 (4.9–7.8)	0.033*	96	5.5 (4.5–6.6)	5.9 (4.5–7.2)	0.402
Creatinine (μmol/L)	96	69.5 (57.0–89.8)	73.5 (60.0–91.5)	0.724	96	74 (62–89)	68 (56–81)	0.206
Ferritin (μg/L)	80	539 (285–1417)	752 (452–1121)	0.385	50	41 (26–82)	41 (29–107)	0.807
**LDH (U/L)**	**73**	**679 (502–955)**	**957 (697–1193)**	0.006**	**48**	**373 (317–405)**	**435 (360–490)**	0.004**
CRP (mg/L)	96	94 (32–150)	115 (86–155)	0.123	97	2 (1–4)	3 (1–6)	0.333
HS-Troponin I (ng/L)	76	7 (4–12)	11 (6–27)	0.014*	48	3 (2–5)	3 (3–5)	0.365
ALT (U/L)	89	32 (22–63)	36 (25–65)	0.510	96	22 (18–31)	21 (13–41)	0.710
Bilirubin (μmol/L)	77	9 (7–12)	13 (8–18)	0.015*	96	9 (7–11)	8 (7–12)	0.984
D-dimer (μg/L)	59	447 (295–811)	548 (407–734)	0.257	85	< 230^&^	< 230^&^	0.769

*Hb* haemoglobin, *WBC* total white blood cell count, *LDH* lactate dehydrogenase, *CRP* C-Reactive protein, HS-Troponin I-High Sensitivity Troponin I, *ALT* alanine transaminase

*p < 0.05

**p < 0.01

^&^230 μg/L lower limit of d-dimer detection

In univariate analysis, baseline serum LDH demonstrated a strong trend of association with increased risk of persistent chest radiograph abnormality but failed to reach statistical significance Hazard Ratio (HR) 1.001, 95% Confidence Interval [95%CI] (1.000–1.005) p = 0.078). However, in a multivariate model correcting for obesity and current or ex-smoking status, baseline serum LDH was a significant predictor for persistent chest radiograph abnormality, HR 1.001 (95% CI 1.000–1.002) p = 0.046.

### Area under the receiver operating characteristic curve (AUROC) analysis

**Fig. 2:**
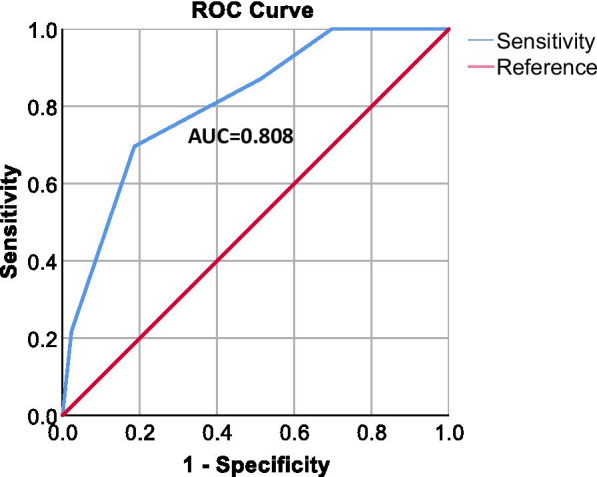
Area under the reciever operator curve (AUROC) for combined persistent chest radiograph risk score. Combined risk score included; Length of Stay (≥ 15 days = 1 point), LDH (≥ 750 U/L = 1 point), Smoking status (current or ex-smoker = 1 point), admission to a level 2 (high dependency) or 3 (intensive care) facility (yes = 1 point) and past medical history of obesity (Body mass index [BMI] > 30 kg/m^2^) (Yes = 1 point). *AUC* area under curve

Using the above identified risk factors we sought to identify a combined variable tool that predicted patients most at risk of developing persistent chest radiograph abnormality at 3-months. Using area under the receiver operating curve (AUROC) analysis, a combined variable analysis was conducted. A combined score with one point each for; length of stay ≥ 15 days, LDH ≥ 750 U/L, positive current or ex-smoking status, admission to a level 2 or 3 care facility, and positive past medical history of obesity (BMI > 30 kg/m^2^) was identified as providing the best prediction model for risk of persistent chest radiograph abnormality with an AUROC of 0.808 (95% CI 0.701–0.915) p < 0.0001 Tables 5, 6 and Fig. 2. With a cut off of 3 points or more demonstrating an AUROC of 0.76 (95% CI 0.625–0.885) with of 0.70 sensitivity and a specificity of 0.81.

**Table 5:** Combined Risk Prediction Score

Variable	Descriptor	Point score
Length of stay	≥ 15 days	1
LDH	≥ 750 U/L	1
Level 2 or 3 care admission	Yes	1
Current or ex-smoker	Yes	1
Obesity (BMI > 30 kg/m^2^)	Yes	1
	Total score	**5**

0–5 point scale with 1 point each for length of stay ≥ 15 days, LDH ≥ 750 U/L, positive current or ex-smoking status, admission to a level 2 (high dependency) or 3 (intensive care) facility and, positive past medical history of obesity [Body mass index (BMI) > 30 kg/m^2^]

**Table 6:** Area under the receiver operating characteristic curve (AUROC) analysis table for risk of persistent chest radiograph abnormality using the combined risk prediction score

Score	Remaining (n)	With score (n)	Persistent abnormality (n)	Resolution (n)	Sensitivity	1-Specificity
0	66	13	0	13	1	1
1	53	11	3	8	1	0.698
2	42	18	4	14	0.87	0.512
3	24	18	11	7	0.697	0.186
4	6	6	5	1	0.217	0.023
5	0	0	0	0	0.000	0.000

0–5 point scale with 1 point each for length of stay ≥ 15 days, LDH ≥ 750 U/L, positive current or ex-smoking status, admission to a level 2 (high dependency) or 3 (intensive care) facility and, positive past medical history of obesity [Body mass index (BMI) > 30 kg/m^2^]

Applying this same risk prediction score to patients outcome destination from the 12-week virtual clinic i.e., discharge (n = 55) or need for a further appointment (n = 46) identified that this risk calculator still performed strongly, predicting patients who needed further follow-up appointments with an AUROC of 0.781 (95% CI 0.664–0.898) p < 0.0001) Table 7.

**Table 7:** Area under the receiver operating characteristic curve (AUROC) analysis table for outcome from virtual 12-week appointment (discharge vs. further follow-up required) using the combined risk prediction score 0–5 point scale with 1 point each for length of stay ≥ 15 days, LDH ≥ 750 U/L, positive current or ex-smoking status, admission to a level 2 (high dependency) or 3 (intensive care) facility and, positive past medical history of obesity [Body mass index (BMI) > 30 kg/m^2^]

Score	Remaining (n)	With score (n)	Further follow-up (n)	Discharged (n)	Sensitivity	1-Specificity
0	63	13	3	10	1	1
1	50	12	2	10	0.900	0.727
2	38	17	6	11	0.833	0.455
3	21	17	13	4	0.600	0.121
4	4	4	4	0	0.167	0.000
5	0	0	0	0	0.000	0.000

## Discussion

In this real world prospective cohort study we observed persistent chest radiograph abnormality in 32% of patients at 12-week follow-up. The risk of persistent abnormality in chest radiographs was found to be increased by extended length of stay, obesity and current or ex-smoking status. Furthermore, in this cohort, patients with persistent chest radiograph abnormality had significantly higher levels of serum LDH at both baseline and follow-up. Using AUROC analysis a 5 point risk assessment score combining length of stay, LDH, smoking-status, level 2 or 3 care admission and obesity was found to strongly predict patients at risk of both developing persistent chest radiograph abnormality, and patients at need of further follow-up appointment.

Our observed rates of persistent chest radiograph abnormality at follow-up are similar to that of Mandal et al. [6] who observe persistent abnormality in 38% of patients at a median of 52 days. Comparing our cohort to that of Mandal et al., our study has both a longer follow-up interval (82 days vs. 52 days) and a higher proportion of patients admitted to critical care (49% vs. 15%), which may account for the similar rates of radiological abnormality despite our longer follow-up interval. In a recently published prospective study Arnold et al. [13] observe 13.6% of patients had persistent chest radiograph abnormality at 3-months. The discrepancey between our results and those of Arnold at el. may be accounted for by only 16% of their cohort being classified has having severe illness compared to 49% of patients admitted to level 2 or 3 care in our study. Consistent with our observation, rates of persistent chest radiological abnormality on CT imaging at 3-month follow-up post hospitalisation with COVID-19, have been estimated at between 40 and 70% [7–9]. Higher rates of detected abnormality on CT analysis would be expected given the increased sensitivity of this modality. It is informative that in the study by Zhao et al. [9] despite the high incidence of persistent CT abnormalities in their study, only 25.4% of the cohort demonstrated impairment in DLCO% predicted suggesting significant parenchymal lung disease. In two studies Tabatabaei et al. [8] and Shah et al. [7] demonstrate that persistent radiological abnormality at 12-week follow-up is associated with increased length of stay [8] and the number of days on oxygen therapy [7]. Further to the findings of these studies we identify that length of stay is an independent risk factor for persistent radiological abnormality post hospitalisation for COVID-19.

We observed significantly higher levels of serum LDH, both at baseline and follow-up, in patients with persistent chest radiograph abnormality post hospitalisation with COVID-19. LDH catalyses the conversion of lactate to pyruvate during glycolysis and is expressed in all human tissues. It is a non-specific marker of tissue damage and has been associated with poor-prognosis in a variety of disease pathologies [14]. In a study of patients with MERS infection, higher peak serum LDH was identified in those with persistent abnormality on chest radiographs at median 42 day follow-up [15]. In patients with COVID-19, elevated LDH has been associated with more severe disease [16] and time to normalisation of LDH has been shown to positively correlate with early CT scan resolution [17].

Using AUROC analysis we identify a five point risk stratification score which strongly predicts risk of persistent chest radiograph abnormality. In our analysis 20% (13 out of 66) patients included in the analysis had a combined risk score of zero and none of these patients were left with persistent chest radiograph abnormalities. We propose that for patients with a score of zero using our AUROC model radiological follow-up post hospitalisation with COVID-19 may not be necessary. This observation requires further validation in a dedicated prospective study.

Our study has a number of limitations which are important to mention. The sample size of our cohort was relatively small (n = 101). However, we present a population with increased level 2 or 3 admission which reflects the real life nature of follow-up prioritisation. Chest radiographs were scored by the clinician assessing the patient and although a standardised proforma was used for scoring chest radiographs, they were not dual reported, so it is not possible to assess concordance between individual assessors. Furthermore, due to the nature of the study it is not definitively possible to ascertain whether the abnormalities on chest radiographs detected at 12-weeks represent complications of COVID-19; or at this point in time, determine what proportion of patients with abnormal radiology will progress to develop significant permanent respiratory pathology. Large long-term prospective follow-up cohort studies such as the currently recruiting PHOSP-COVID study (ISRCTN10980107) will hopefully provide insight into this as time progresses. As our cohort was developed in ‘real-time’ as the pandemic evolved and tests completed reflected standard-of-care procedures at UHSFT, paired data for LDH is not available for all patients in the cohort. However early in the COVID-19 pandemic an institution-wide COVID-19 blood panel was employed at UHSFT for all suspected and confirmed admissions with COVID-19 and later for patients in the post COVID-19 follow-up clinic. Consequently pathology tests were not requested in an unselected manner by the assessing clinician. Hence it is unlikely that the lack of available LDH data for some patients resulted in a selection bias in our analysis.

## Conclusion

In this real world cohort of hospitalised patients with COVID-19, we identify that persistent chest radiograph abnormality was present in 32% of patients. Serum LDH, length of stay, obesity and current or ex-smoking status were identified as risk factors for persistent radiological abnormality. In AUROC analysis a five point composite model strongly predicted persistent chest-radiograph abnormalities. This tool could be used to stratify patients at greatest need of radiological follow-up and those in which it may not be required. These observations require further validation in a dedicated prospective study.

## Supplementary Information

**Additional file 1.** 1)** Supplemental Figure 1**. CONSORT flow diagram of the cohort. 2)** Supplemental Table 1**. Summary results table for pulmonary function tests completed at the time of writing for the whole cohort and comparison between those with complete resolution (complete resolution in chest radiograph at 12-weeks) versus those with persistent abnormality (persistent chest radiograph abnormality at 12-weeks). 3) Supplemental information with description of patterns of pulmonary fibrosis identified on Chest computed tomography (CT) scanning.

## Data Availability

All data generated or analysed during this study are included in this published article [and its supplementary information files].

## Abbreviations

95%CI: 95% Confidence interval
AP: Anteroposterior
AUROC: Area under the receiver operating characteristic curve
BMI: Body mass index (kg.m^−^^2^)
CONSORT: Consolidated Standards of Reporting Trials
COPD: Chronic obstructive pulmonary disease
COVID-19: Coronavirus disease 2019
CT: Computerised tomography
CXR: Chest radiograph
HR: Hazard ratio
IQR: Interquartile range
LDH: Lactate dehydrogenase
Level 2: High dependency facility
Level 3: Intensive care facility
LOS: Length of stay
MERS: Middle east respiratory syndrome
SARS: Severe acute respiratory syndrome
SARS-CoV-2: Severe acute respiratory syndrome coronavirus 2
PA: Posterioanterior
PCR: Polymerase chain reaction
POCT: Point-of-care testing
UHSFT: University Hospitals Southampton NHS Foundation Trust
