# Supplementary material for: Risk factors for persistent abnormality on chest radiographs at 12-weeks post hospitalisation with PCR confirmed COVID-19
Source: Respir Res. 2021 May 21;22:157. doi: 10.1186/s12931-021-01750-8 (PMC8139368; doi:10.1186/s12931-021-01750-8)
Supplement: Supplementary file 1 — Additional file 1. 1) Supplemental Figure 1. CONSORT flow diagram of the cohort. 2) Supplemental Table 1. Summary results table for pulmonary function tests completed at the time of writing for the whole cohort and comparison between those with complete resolution (complete resolution in chest radiograph at 12-weeks) versus those with persistent abnormality (persistent chest radiograph abnormality at 12-weeks). 3) Supplemental information with description of patterns of pulmonary fibrosis identified on Chest computed tomography (CT) scanning. [file 12931_2021_1750_MOESM1_ESM.docx]

**Online Data Supplement**

**Risk factors for persistent abnormality on chest radiographs at 12-weeks post hospitalisation with PCR confirmed COVID-19.**

**Authors** TJM Wallis^1^, E Heiden^2^, J Horno^2^, B Welham^3^, H Burke^3^, A Freeman^3^, L Dexter^2^, A Fazleen^3^, A Kong^1^, C McQuitty^1^, M Watson^2^, S Poole^4^, NJ Brendish^5^, TW Clark^4^, TMA Wilkinson^1^, MG Jones^1*^ and BG Marshall^1*^

*Joint Last Author

Corresponding Author: Dr Tim Wallis NIHR Southampton Biomedical Research Centre Research Fellow. MP218 D-Level South Academic Block University Hospital Southampton SO16 6YD. email timothy.wallis@soton.ac.uk

**Supplemental Materials**

**Supplemental Figure 1**

**
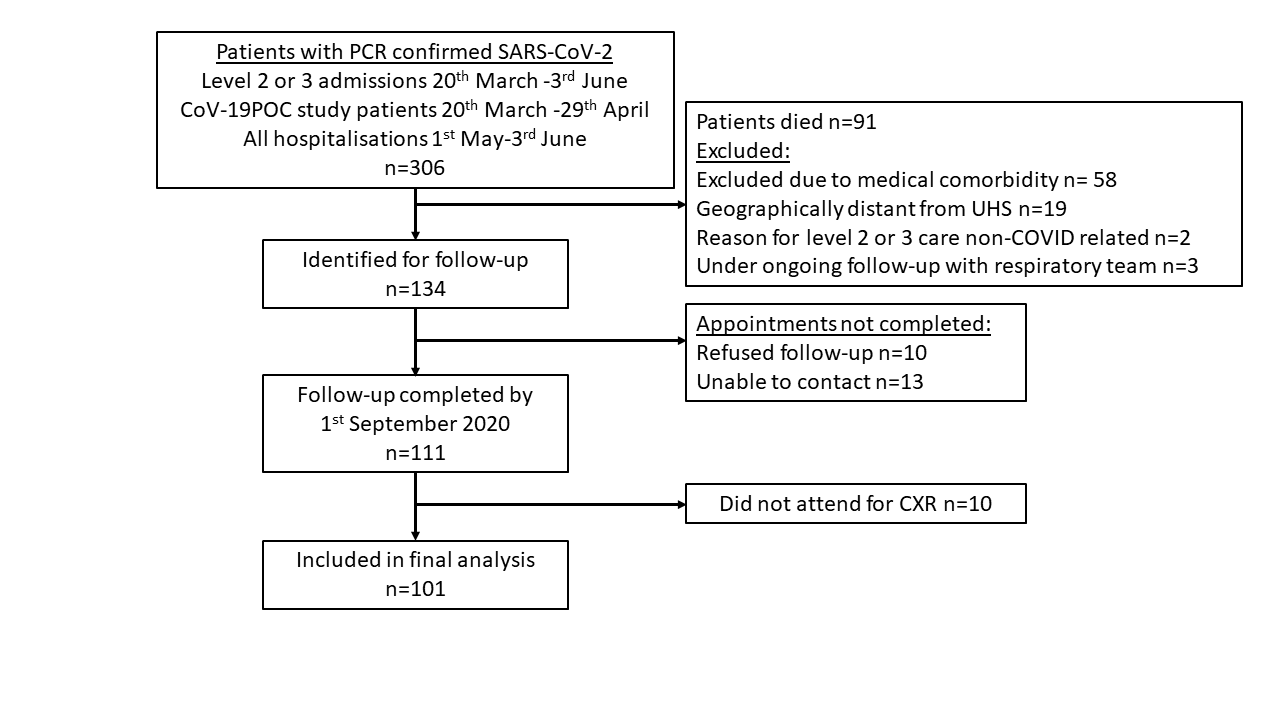
**

**Supplemental Figure 1** Consolidation Standards of Reporting Trials (CONSORT) Figure (1) for the cohort. UHS University Hospital Southampton Foundation Trust. All patients screened had PCR confirmed SARS-CoV-2 infection. Level 2 (High Dependency Facility), Level 3 (Intensive Care Facility) CXR- Chest Radiograph.

**Supplemental Information**

**Supplemental Table 1**

|  | **Whole Cohort**  n=101  Median (IQR) or n (%) | **Complete Resolution**  n=69  Median (IQR) or n (%) | **Persistent Abnormality** n=32  Median (IQR) or n (%) | **p value** |
| --- | --- | --- | --- | --- |
| **Patients with PFT** | 36 (36%) | 19 (28%) | 17 (53%) |  |
| **PFT Interval (weeks)** | 26 (22-29) | 27 (24-33) | 25 (21-28) | 0.103 |
| **FEV1 (%)** | 84 (74-102) | 87 (80-108) | 79 (72-94) | 0.076 |
| **FVC (%)** | 85 (71-101) | 93 (79-111) | 74 (69-93) | 0.057 |
| **TLCO (%)** | 81 (75-93) | 85 (78-96) | 77 (67-85) | 0.071 |

**Supplemental Table 1**. Summary results table for pulmonary function tests completed at the time of writing for the whole cohort and comparison between those with complete resolution (complete resolution in chest radiograph at 12-weeks) versus those with persistent abnormality (persistent chest radiograph abnormality at 12-weeks). PFT= pulmonary function test, IQR= interquartile range. FEV1 (%) = forced expiratory volume in 1 second percent predicted. FVC (%) = forced vital capacity percent predicted. TLCO (%) = Transfer coefficient of the lung for carbon monoxide percent predicted. P values compare complete resolution vs. persistent abnormality, assessed using Mann-Whitney U test.

**Patients with pulmonary fibrosis identified on Chest computed tomography (CT) scanning**

12 patients with persistent chest x-ray abnormality had fibrosis identified on follow-up Chest CT of these; nine received invasive mechanical ventilation (IMV), in addition one of these also underwent Extracorporeal membrane oxygenation (ECMO), one patient received CPAP/NIV and two patients received supplemental oxygen therapy only

**Patient 1**

Highest Respiratory Support: IMV

CT Appearances: Multifocal scarring with traction bronchiectasis

**Patient 2**

Highest Respiratory Support: IMV

CT Appearances: Multifocal consolidation and ground glass opacification

**Patient 3**

Highest Respiratory Support: IMV

CT Appearances: Non-specific pattern of course subpleural and peri-bronchial pulmonary fibrosis with irregular septal thickening and traction bronchiectasis but without honeycombing. Minor ground-glass component.

**Patient 4**

Highest Respiratory Support: IMV

CT Appearances: Bilateral, apical predominant ground-glass opacification with mild/moderate peri-bronchial and subpleural fibrosis with traction bronchiectasis, reticulation and irregular septal thickening but no honeycombing.

**Patient 5**

Highest Respiratory Support: ECMO

CT Appearances: Extensive bilateral interlobular septal thickening, atelectasis and ground-glass opacification.

**Patient 6:**

Highest Respiratory Support: IMV

CT Appearances: Peripheral upper lobe predominant linear subpleural fibrosis without honeycombing

**Patient 7**:

Highest Respiratory Support: Oxygen

CT Appearances: subpleural ground glass attenuation and reticulation minor traction bronchiectasis no honeycombing.

**Patient 8:**

Highest Respiratory Support: Oxygen

CT Appearances: Mild Ground-glass opacification and subpleural consolidation

**Patient 9:**

Highest Respiratory Support: IMV

CT Appearances: upper and mid zone reticulation and septal thickening

**Patient 10**

Highest Respiratory Support: CPAP/NIV

CT Appearances: mild bibasal subpleural reticulation and traction bronchiectasis

**Patient 11**

Highest Respiratory Support: IMV

CT Appearances: Bilateral patchy ground glass opacification and traction bronchiectasis

**Patient 12**

Highest Respiratory Support: IMV

CT Appearances: Bilateral middle and lower lobe fine fibrosis and traction bronchiectasis

**References**

1. Rennie D. CONSORT revised--improving the reporting of randomized trials. Jama. 2001;285(15):2006-7.
